# Rewiring of the Liver Transcriptome across Multiple Time-Scales Is Associated with the Weight Loss-Independent Resolution of NAFLD Following RYGB

**DOI:** 10.3390/metabo12040318

**Published:** 2022-04-02

**Authors:** Peng Lei, Chijioke Chukwudi, Prabh R. Pannu, Shijie He, Nima Saeidi

**Affiliations:** 1 Division of General and Gastrointestinal Surgery, Department of Surgery, Massachusetts General Hospital, Harvard Medical School, Boston, MA 02114, USA; plei2@mgh.harvard.edu (P.L.); cchukwudi@mgh.harvard.edu (C.C.); ppannu@mgh.harvard.edu (P.R.P.); she9@mgh.harvard.edu (S.H.); 2 Center for Engineering in Medicine, Department of Surgery, Massachusetts General Hospital, Harvard Medical School, Boston, MA 02114, USA; 3 Shriners Burn Hospital for Children, Boston, MA 02114, USA

**Keywords:** RYGB, NAFLD, RNA sequencing, systems biology, time course study, graph network

## Abstract

Roux-en-Y gastric bypass (RYGB) surgery potently improves obesity and a myriad of obesity-associated co-morbidities including type 2 diabetes and non-alcoholic fatty liver disease (NAFLD). Time-series omics data are increasingly being utilized to provide insight into the mechanistic underpinnings that correspond to metabolic adaptations in RYGB. However, the conventional computational biology methods used to interpret these temporal multi-dimensional datasets have been generally limited to pathway enrichment analysis (PEA) of isolated pair-wise comparisons based on either experimental condition or time point, neither of which adequately capture responses to perturbations that span multiple time scales. To address this, we have developed a novel graph network-based analysis workflow designed to identify modules enriched with biomolecules that share common dynamic profiles, where the network is constructed from all known biological interactions available through the Kyoto Encyclopedia of Genes and Genomes (KEGG) resource. This methodology was applied to time-series RNAseq transcriptomics data collected on rodent liver samples following RYGB, and those of sham-operated and weight-matched control groups, to elucidate the molecular pathways involved in the improvement of as NAFLD. We report several network modules exhibiting a statistically significant enrichment of genes whose expression trends capture acute-phase as well as long term physiological responses to RYGB in a single analysis. Of note, we found the HIF1 and P53 signaling cascades to be associated with the immediate and the long-term response to RYGB, respectively. The discovery of less intuitive network modules that may have gone overlooked with conventional PEA techniques provides a framework for identifying novel drug targets for NAFLD and other metabolic syndrome co-morbidities.

## 1. Introduction

The discovery of novel druggable targets for metabolic diseases, such as type 2 diabetes, obesity, and non-alcoholic fatty liver disease (NAFLD), has recently been fueled by the application of multi-omics technology [1] to elucidate the metabolic and signaling mechanisms of disease progression and amelioration. For example, Albrechtsen et al. recently demonstrated the application of LC/MS-based proteomics on plasma samples derived from patients who underwent Roux-en-Y Gastric Bypass (RYGB) to discover secreted proteins involved in chronic inflammation that correlate with the beneficial metabolic effects of the surgery, namely weight loss and improved insulin sensitivity [2]. RYGB and sleeve gastrectomy are the most potent and long-term treatments available for obesity [3], and lead to significant improvement in a large number of obesity-associated sequalae, including complete resolution of NAFLD [4]. Therefore, understanding the molecular mechanisms through which RYGB rewires liver function may lead to the identification of novel therapeutic targets for metabolic syndrome driven indications.

Time-series omics data are particularly useful for studying the dynamic behavior of biological systems and provide novel insight into disease progression and amelioration at the molecular level. However, conventional -omics analysis workflows often fall short for such data because they implicitly rely on pair-wise statistical comparisons, either between two experimental conditions at a single time-point, or between two time-points under one experimental condition, neither of which effectively capture the underlying molecular mechanisms spanning across multiple timescales. Several methods have been previously developed to address this and are based on mathematical and statistical models, as varied as Spline regression [5], Bayesian networks [6], Gaussian processes [7], Impulse modeling [8], and Fourier Transforms [9] to perform differential expression analysis or, expression profile clustering to reveal co-expressed trajectories. However, these methodologies treat biomolecules (e.g., transcripts) as purely mathematical entities, and do not incorporate known biological connections between them based on metabolic reactions, signaling cascades, or transcriptional regulation.

We herein present a novel graph network-based analysis workflow designed to identify modules enriched with biomolecules that share common dynamic profiles, where the network is constructed from all known biological interactions available through the KEGG resource. Focusing on time-series RNA-seq data, we studied the impact of rodent RYGB on liver metabolic and signaling pathways over the course of three months, with the intent of unraveling a dynamic liver remodeling that correlates with the beneficial metabolic effects of the surgery. Our results show that genes with similar Log_2_ fold change (L2FC) vector trajectories between RYGB and weight-matched (WM) control groups tend to significantly cluster in nodal proximity on a large-scale network comprising human biological pathways, suggesting that functionally related genes share common expression dynamics. Our method reveals less-intuitive pathway modules activated by RYGB across different time points, thus facilitating the discovery of novel hepatic drug targets for metabolic syndrome indications.

## 2. Results and Discussion

**Figure 1:**
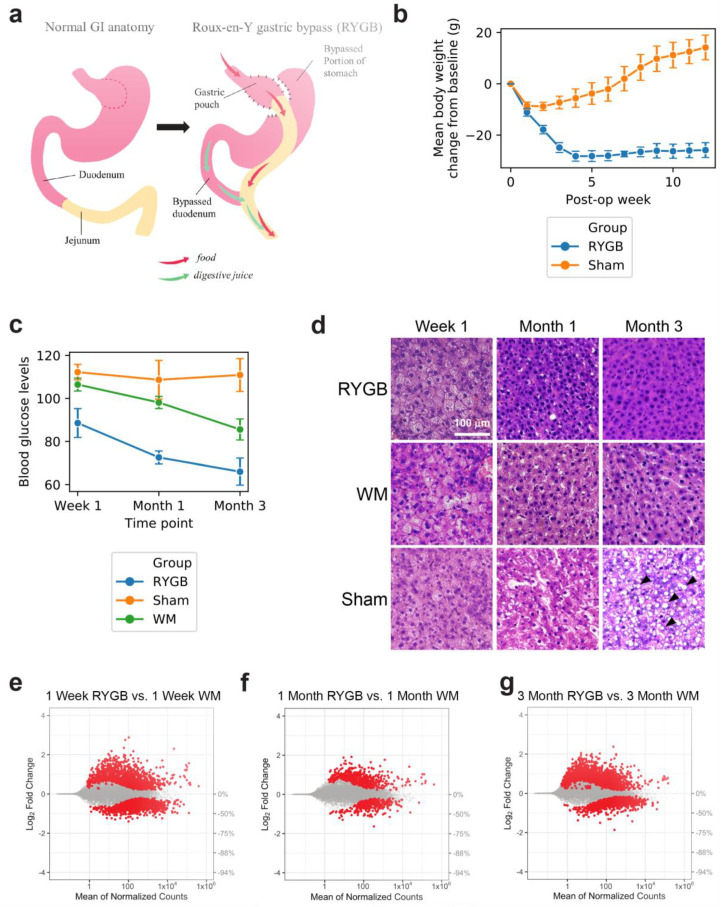
(**a**) Schematic of RYGB surgery. (**b**) Comparison of mean body weight change from baseline between Sham and RYGB groups at one day intervals for 12 weeks. (**c**) Comparison of plasma glucose levels (mg/dl) between RYGB, Sham, and weight-matched (WM) groups at 1 week, 1 month, and 3 month time points. (**d**) Histological analysis of liver slices for RYGB and both controls (Sham, WM) at 1 week, 1 month, and 3 month time points. Several of the lipid droplets are identified by the arrowheads; (**e**–**g**) MA plots showing gene expression Log_2_ fold change between RYGB and WM groups at each time point for all transcripts measured using RNA-seq.

RYGB or Sham surgery was performed on diet-induced obese (DIO) male Sprague Dawley rats. Immediately after the operations, the Sham-operated animals were further split into two cohorts wherein one group had an ad libitum access to food (i.e., Sham cohort) and the second group was calorie restricted to provide WM cohort (please see Methods). Animals were sacrificed post-operatively at week 1, month 1, and month 3. Five animals were used for each experimental group and each time point. These specific timepoints were selected to provide a comprehensive view of the temporal dynamics of the metabolic and physiological adaptations to the surgery. More specifically, while the 1 week timepoint corresponds to the immediate post-operative effects, at around 1 month after surgery, many of the physiological changes (e.g., body weight and insulin sensitivity) start stabilizing. By 3 months postoperatively, the impacts of surgery are well established and stabilized, and therefore this timepoint was used as a surrogate for mechanisms involved in the sustained and long-term effects of the surgery [10]. We first show that RYGB (Figure 1a, Methods) causes significant body weight reduction that is sustained over the three months duration of the study. In contrast, after a short period of weight loss immediately after the operation, sham-operated animals continue to gain weight (Figure 1b). Blood glucose levels in the RYGB animals decrease steadily following surgery, while for the sham animals they remain unchanged, and in the weight-matched cohort we observed a less dramatic decrease in blood glucose levels (Figure 1c). H&E staining was performed to evaluate changes in the liver morphology and lipid content, demonstrating no significant differences between the RYGB and weight-matched (WM) groups at post-operative week 1. However, at 1 and 3 months after surgery, there is a significant reduction in the lipid content of RYGB-operated animals compared to both sham and WM animals (Figure 1d). Importantly, the observed differences between RYGB and WM animals indicates that improvement in NAFLD is mediated via mechanisms extending beyond caloric restriction and weight loss. RNA-seq was then applied on whole rat liver at 1 week, 1 month and 3 months following rodent RYGB, sham surgery, or sham surgery with calorie restriction (i.e., WM group), to measure 23,113 transcripts and capture gene expression changes across all liver cell types. The RNAseq was performed on the sample individually (i.e., no sample pooling was performed). Genes whose corresponding transcripts were denoted as statistically significant based on the Benjamini-Hochberg-corrected *p*-value threshold of 0.05 were considered differentially expressed between RYGB and any of the two control groups, and the complete list can be found in Supplemental Table S1. In this study we primarily focus on the comparison between RYGB and WM control groups, as that would identify weight loss-independent effects of the surgery. However, the data related to sham animals is provided in Supplemental Table S2. Gene dysregulation in RYGB vs. WM controls for each time point is shown in Figure 1e–g with the greatest levels of gene expression dysregulation observed at 3 months post-surgery.

**Figure 2:**
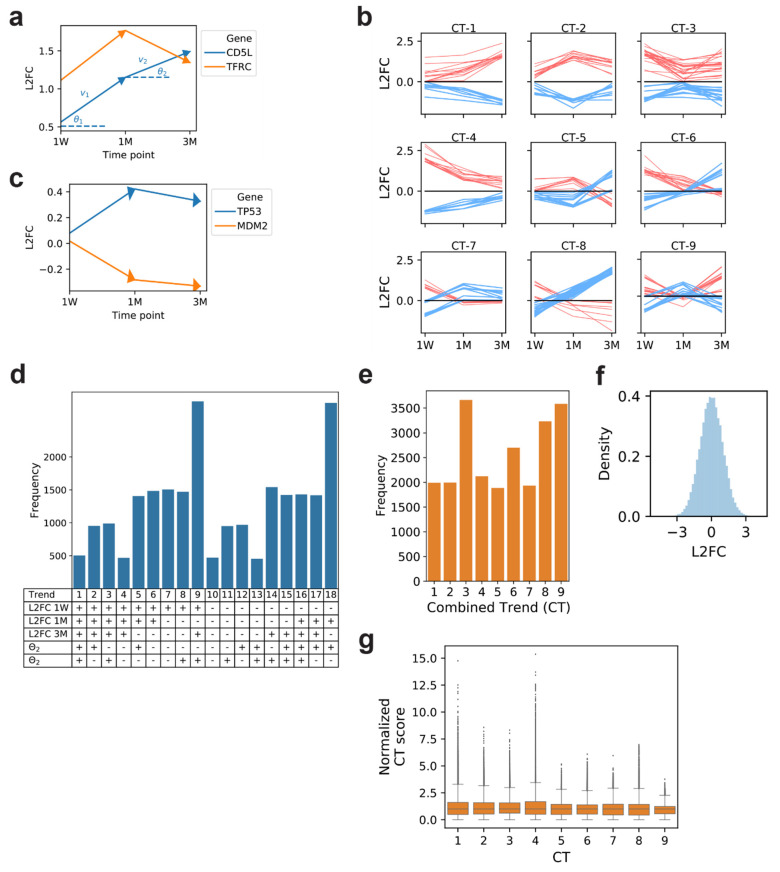
(**a**) Example of a dynamic trend plot (DTP) for two genes showing the Log_2_ fold-change (L2FC) between RYGB and WM groups at the 1 week, 1 month, and 3 month time points. (**b**) DTP of the top 1% scoring genes designated to each combined trend (CT) based on mean L2FC values. (**c**) Example for the DTP of two genes where one (*MDM2*) is a negative regulator of the other (*TP53*). (**d**) Frequency plot showing the number of genes that are classified as belonging to one of 18 possible trends based on the mean L2FC at each time point. (**e**) Frequency of genes classified as belonging to one of 9 possible combined trends (CT) where a CT represents the two original dynamic trends that are mirror images of each other along the x-axis. (**f**) Distribution of L2FC values between RYGB and WM at all time points. (**g**) Distribution of the normalized CT score for each CT.

To analyze time-series trends in gene expression, we recognize that the dynamics in L2FC between RYGB and WM groups for each gene at three time points can be represented as two vectors, ${\vec{v}}_{1}$ and ${\vec{v}}_{2}$, where *θ*_1_ and *θ*_2_ represent directions between the corresponding vector and the horizontal axis (Methods, Figure 2a). For example, the dynamic trend plot (DTP) for *CD5L*, a negative regulator of lipid synthesis, shows a positive L2FC between RYGB and WM at all three time points and a steady increase from 1 week to 3 months, as reflected by positive *θ*_1_ and *θ*_2_, while the DTP for *TFRC*, an iron uptake transporter, trends similarly from 1 week to 1 month, but exhibits a subsequent decrease from 1 month to 3 month captured by a negative *θ_2_* (Figure 2a). We determined 18 possible trends capturing the dynamics of gene expression fold changes across three time points (Methods) and combined trends whose DTP trajectories are mathematical mirror images across the x-axis, under the premise that negatively co-regulated genes may exhibit the same biological signal in opposite directions, thus resulting in nine combined trends (CTs, Figure 2b). The frequency of genes classified in each of these 18 distinct trends and 9 CTs based on mean expression levels are displayed in Figure 2d, and 2e respectively. It is important to note that when L2FC values are randomly assigned from the 23,112 genes at three time points, the frequency distribution of trends is not uniform along the CTs (Supplemental Figure S1). This is intuitive since the distribution of L2FC values (Figure 2f) has a mean close to zero with relatively high kurtosis. As such, the likelihood of gene expression values fluctuating around zero, where the L2FC alternates between positive and negative values across the three time points, as dictated by CT-9, is higher than other trends. CT-1, in contrast, requires that the DTP increasingly moves away from zero across the time course, which is statistically less likely to occur.

We recognize that animal-to-animal variation in normalized gene expression counts at any given time point leads to variation in L2FC values between RYGB and control groups. Therefore, each gene is further assigned a discrete probability distribution (DPD) of possible CTs (Methods). As a motivating example, *TP53*, a known tumor suppressor gene, is negatively regulated through interaction with oncoprotein *MDM2* [11] and these two genes exhibit CT-1 and CT-2 behavior, respectively, based on their mean L2FC values (Figure 2c). However, the inherent variation in expression of *TP53* at 3 months suggests that the gene could exhibit CT-1 dynamics within a margin of error. The DPD for each gene is determined by Monte-Carlo sampling expression counts at each time point, modeled by a log-normal distribution using the mean and standard deviation of normalized expression counts at each time point, from which L2FC values and corresponding CTs are computed. The fraction of cases from the Monte-Carlo sampling for which each CT was observed constitutes the DPD of a gene. In this regard, a CT and CT-score is assigned for each gene, the latter being the fraction of simulated cases that results in the assigned CT (Supplemental Table S1). For *TP53*, since the DPD shows that 24.3% of the simulated cases result in CT-1 dynamics and 23.0% for CT-2, thereby suggesting that the likelihood of *TP53’s* true expression trajectory as CT-1 is equally as likely as CT-2. Each CT-score is then normalized to the median value of all the CT-scores for the specified CT to obtain a normalized CT- score (NCTS). The distribution of NCTS values for genes in each trend is shown in Figure 2g.

**Figure 3:**
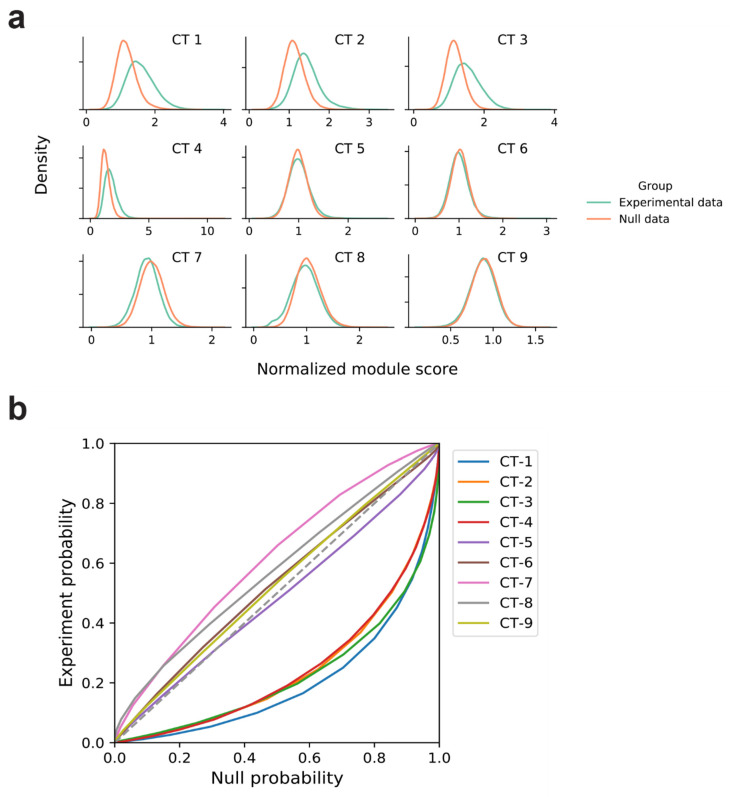
(**a**) Distribution of normalized module scores for the case where experimental RNAseq data for RYGB vs. WM liver samples compared to the control case where L2FC values for all time points are shuffled. (**b**) Probability–probability plots comparing the experimental and control cases for each CT.

We then used a bipartite graph network encompassing 337 metabolic and signaling pathways provided by the KEGG [12] resource (Methods) to discover network modules with high average NCTS, which highlighted a cluster of genes with nodal proximity that share common expression dynamics across three time points. For each sub-network obtained, a module CT score (MS) is computed for each CT by taking the mean NCTS for a given CT across the genes in the module. Significance of high MS is determined relative to distributions of MS for that CT obtained from a null model where all L2FC values for all genes and time points are shuffled. We report that for CTs-1–4, the MS are higher when computed with actual expression data derived from RYGB vs. WM conditions than when using random expression data (Figure 3a). A probability–probability plot of the likelihood of observing a score or lower in each of the MS distributions shows that CTs-1–4 are significantly divergent from the null model in that they have more modules with statistically higher scores (Figure 3b). This is supported by Kologorov–Smirnov tests comparing the distributions of MS to the null model for each CT (Supplemental Table S3). We compute for each module the probability of obtaining a module with a MS as high as what was observed by random chance, denoted as ρ. The top scoring modules for CTs-1–4, for which the adjusted ρ (Benjamini–Hochberg corrected) is less than the false discovery rate of 0.05, are deemed as significantly enriched in genes of a certain CT.

**Figure 4:**
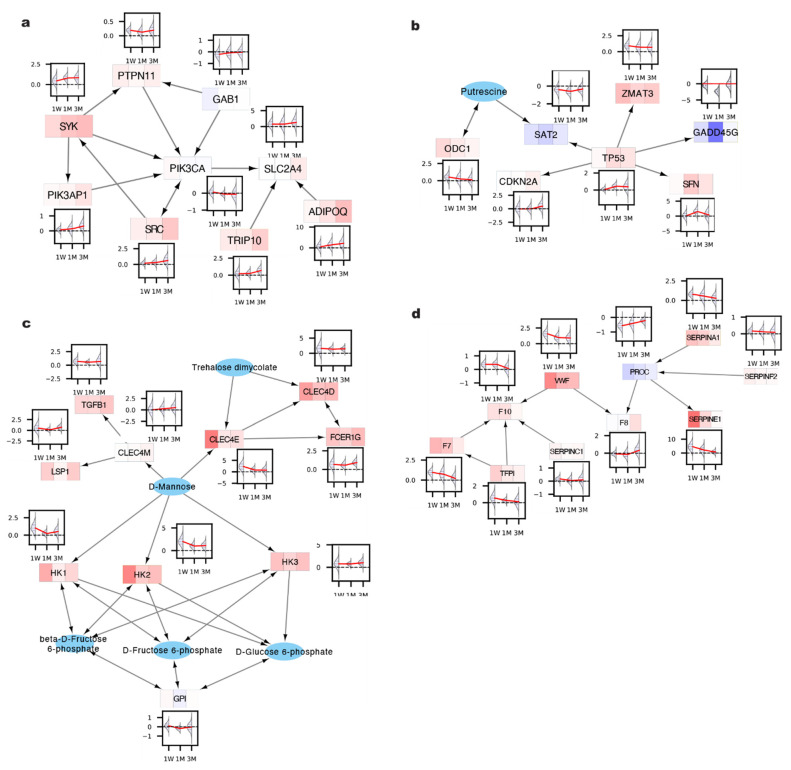
Sample modules enriched for genes exhibiting (**a**) CT-1, (**b**) CT-2, (**c**) CT-3, and (**d**) CT-4 dynamic behavior.

For each CT, we report a representative significantly enriched module (Figure 4) while all other modules and their MS scores are available in Supplemental Table S4. Focusing on CT-1 behavior and rank-sorting modules based on the density of CT-1 genes, we identified a module featuring the genes *SRC, SYK, PIK3AP1, PIK3CA, SLC2A4* (or *GLUT4*)*, TRIP10, ADIPOQ, PTPN11,* and *GAB1*, where the L2FC between RYGB and WM either starts as positive at week 1 and continues to increase with time or starts as negative and continues to decrease (Figure 4a). The genes included in this module are primarily involved in the PI3K/AKT/GLUT4 signaling pathway which is a master regulator of cell metabolism [13]. In the context of RYGB, CT-1 behavior represents biological rewiring as a consequence of the surgery that is sustained long-term over the course of three months, which coincides with its sustained metabolic benefits. In this case, we show that *TRIP10* and *ADIPOQ* gene expression increases over time in RYGB relative to WM. *TRIP10* is required for the GLUT4 translocation to the plasma membrane in response to insulin [14], and its continuous increase would reflect an improved hepatic insulin sensitivity and systemic glycemic control following RYGB. *ADIPOQ,* encoding adiponectin, is a known adipokine with well-characterized anti-diabetic properties [15]. Importantly, hepatic-specific expression of adiponectin has shown to be inversely correlated with NAFLD disease-state [16]. While the function of these two genes in the context of metabolic phenotypes has been well characterized, their connection to the broader PI3K-AKT signaling pathway, which also regulates GLUT4 function, remains unclear. The phosphorylation and subsequent activation of PK3AP1 and PIK3CA by SYK, whose CT-1 gene expression score was highest in this module, is generally discussed in an oncogenic context [17], but may also play a role in long-term glucose homeostasis post RYGB. The identification of this module also highlights the benefit of our algorithm in its ability to discover statistically significant dynamic trends in gene clusters that may not have been identified using conventional PEA analysis, which would involve three binary comparisons between RYGB and WM with several genes not meeting conventional L2FC or *p*-value thresholds at individual time points, oftentimes due to the low sample size constraints of animal experiments.

Between the 1 week and 1 month time points, CT-2 shares a similar dynamic trend as CT-1; however, CT-2 genes peak in the absolute value of L2FC between RYGB and WM at one month and subsequently decrease or plateau by three months, suggesting that the impact of the surgery on these liver genes coincides with macroscopic phenotypic changes that manifest at one month and either sustain or wane off at future time points, such as hepatic lipid content clearance (Figure 1c). We report a module containing genes *TP53, SFN, SAT2, ZMAT3, CDKN2A, SIRT1, FOXO3, CCNB3, CDK1, PCK1,* and *ODC1*, with the first four listed genes exhibiting the highest CT-2 dynamics. TP53 signaling has been shown to be a potent regulator of lipid metabolism [18] and its pharmacological activation ameliorates NAFLD [19]. The pathway’s activity plateaus from 1 month to 3 months, based on the expression profile of downstream targets *SFN, SAT2,* and *ZMAT3*, suggesting that once the stored lipid is cleared out during the first month (potentially through stimulation of fatty acid oxidation [19]) the liver does not require further up-regulation of these genes. The fact that a transcription factor and its numerous downstream targets share the same dynamic trend validates the premise of our work that sub-networks of connected genes are more likely regulated by a common mechanism. Moreover, observing expression data solely at the 1-week time point may have drawn concern about the L2FC of *TP53* between RYGB and WM, mutations of which are observed in 50% of all cancers [20]. However, analyzing the full time-series trend instead is advantageous to the investigator recognizing that there is long-term rise in P53 signaling which may contribute to the significant protective effects of RYGB against liver cancer [21].

We also report modules whose dynamics reflect an acute phase response at 1 week as a result of RYGB, which is captured by the expression dynamics of CT-3 and CT-4. For CT-3, we highlight a module (Figure 4c) with predominantly fructose metabolism genes (*MPI, TKT, HK3, HKDC1, PFKM, TIGAR, PFKFB3, HK2, PFKP, PFKL, HK1, HIF1A)* whose L2FC between RYGB and WM starts high, decreases at 1 month, and subsequently increases again by 3 months. This phenomenon might be a consequence of glucose homeostasis being regulated by several triggers across multiple time scales and that an escalation of fructose metabolism genes can occur both acutely after RYGB, as well as after 1 month to achieve a long-term steady state. Lastly, CT-4 gene expression is similar to CT-3, except the linear trend between 1 month and 3 month matches that between 1 week and 1 month and we report a module (Figure 4d) predominantly comprised of genes encoding secreted proteins functioning in the coagulation cascade pathway. Hyperactivation of coagulation cascade and wound healing shortly after RYGB may be a general response to an invasive procedure, a response that gradually dwindles over three months. The long-term reduction in the coagulation factors in RYGB compared to WM is particularly of interest as obesity is characterized as a hypercoagulative state, where the increase in coagulation factors has been linked to many of obesity-related comorbidities such as T2DM and NAFLD [22].

## 3. Materials and Methods

### 3.1. Animals

The animal experiments and animal care were performed in compliance with and were approved by the Institutional Animal Care and Use Committees of the Massachusetts General Hospital, Boston, MA (PHS Assurance Number D16-00361). Diet-induced obese (DIO) male Sprague Dawley rats (Charles River Laboratories, Wilmington, MA, USA) were used for all studies. Obesity was induced by feeding the animals ad libitum with a high-fat diet (HFD), which provides 60% of total energy as fat, 20% as carbohydrate, and 20% as protein (D12492 diet, Research Diets Inc., New Brunswick, NJ, USA). At the time of surgery, DIO rats weighed 675 ± 25 g. Animals were individually housed and were maintained on 12-h light, 12-h dark cycle (lights on at 0700 h) in facilities with an ambient temperature of 19–22 °C and 40–60% humidity. For each experimental group and each time point, five animals were used.

### 3.2. Roux-en-Y Gastric Bypass (RYGB) and Sham Surgery

The RYGB procedure was performed according to the method previously described [23]. The total length of the small intestine was measured, the ligament of Treitz was identified, and the jejunum was transected approximately 10 cm downstream of this ligament. An end-to-side jejuno-jejunostomy and a gastrojejunostomy were created. A pouch was created by transecting the stomach. The laparotomy was closed with a 5-0 polydioxanone (PDS) suture in two layers. The sham operation consisted of laparotomy, jejunal transection, and repair; and was performed in animals that were age- and weight- matched preoperatively to animals undergoing RYGB. To generate the WM group, their daily food intake was adjusted such that their daily percent body weight loss matched with the RYGB group.

### 3.3. Intraperitoneal Glucose Tolerance Test

Animals were fasted overnight. The following morning, animals were weighed, and a glucose solution (1 g glucose/kg body weight) was administered intraperitoneally. Blood glucose levels were measured before glucose administration (time 0) and 10, 20, 30, 45, 60, and 120 min after intraperitoneal glucose administration using a blood glucose meter via tail vein.

### 3.4. RNA-seq

RNA-seq was performed for each sample individually (i.e., no sample pooling was performed). The sequencing was used to measure transcript counts corresponding to 23,113 genes in crushed liver samples from which differentially expressed genes were determined for RYGB vs. control groups (i.e., Sham or WM) at 1 week, 1 month, and 3 months post-surgery. RNA was isolated from approximately 60 mg homogenized tissue using TRIzol reagent, and RNA quality was subsequently determined with Tapestation 4200 RNA ScreenTape analysis. A total of 500 ng of RNA was used for sequencing after ribosomal RNA depletion using the Illumina RiboZero rRNA Removal Kit. AMPure XP paramagnetic beads were utilized for RNA purification and library clean-up of contaminants throughout protocol. The Illumina TruSeq Stranded mRNA Library Prep kit was used for library preparation, comprised of fragmentation, and priming, 1st strand cDNA synthesis, 2nd strand DNA synthesis, followed by clean up with AMPure XP beads, according to manufacturer’s protocol. A-tailing and adaptor ligation was performed with TruSeq RNA Single Indexes and followed by two additional clean ups with AMPure XP Beads. PCR enrichment was followed by additional clean up with AMPure XP Beads. Libraries were normalized and pooled. The Tapestation 4200 was used to analyze library quality, which had an average peak size of 323 bp. The libraries were sequenced on the Illumina NextSeq 550 v2.0 kit. DESeq2 [version 1.15] package in R was used to compute the log_2_ fold change and Wald test *p*-value for each transcript at each time point. Genes whose corresponding transcripts were denoted as statistically significant based on the Benjamini–Hochberg-corrected *p*-value threshold of 0.05 were considered differentially expressed in RYGB compared to control animals. The complete list of DEGs can be found in Supplemental Table S1.

### 3.5. Combined Trends

Combined trends (CTs) are defined by first identifying all theoretical gene expression trajectories in the L2FC vs. time coordinate space based on the RYGB vs. WM comparison at each time point. A gene expression trajectory is comprised of two vectors, ${\vec{v}}_{1}$ and ${\vec{v}}_{2}$, with directions *θ*_1_ and *θ*_2_, that capture the mean expression L2FC dynamics between 1 week and 1 month post-surgery, and 1 month and 3 months post-surgery respectively. While mathematical combinatorics of assigning a positive or negative sign to each of the three L2FC values would suggest 32 theoretical trends, only 18 of them are feasible after recognizing that vectors crossing the x-axis necessitate specific *θ* values. For example, if the signs of the L2FC vector for a particular gene were determined to be [+,−,+], *θ*_1_ and *θ*_2_ need to be negative and positive respectively. These 18 possible trends were further reduced to 9 CTs by combining trends with L2FC trajectories that were reflections of each other over the x-axis.

### 3.6. Gene CT Scores

Animal-to-animal variation in normalized gene expression counts implies that the L2FC between RYGB and WM groups is a random variable, whose underlying distribution results in a corresponding distribution of CTs that describe the gene expression trajectory. To discriminate between higher and lower confidence gene CT assignments, we determine the probability of observing each CT based on the discrete probability distribution (DPD) of possible CTs, rather than relying on a single trend derived from the mean L2FC values. To generate a gene’s DPD, we first model the normalized expression count distribution for each sample group (condition and time-point) by a log-normal probability density function (PDF) using the mean and standard deviation of the observed normalized counts as parameters. We then applied Monte-Carlo sampling from the each of the 6 PDFs generated for each gene 1000 times, computing L2FC between RYGB and WM at each time point, along with the corresponding expression trajectory-based CT. For each CT, the gene CT score is defined as the fraction of sampling cases where the CT was observed. In order to compare scores across CTs, we normalized the gene CT scores to the median CT score across all genes, resulting in the normalized gene CT score. This normalization was necessary because genes are more likely to be assigned to certain CTs, such as CT-9, over others in the scenario where all expression values are derived from the same underlying distribution, which causes an inflation in gene scores for CTs that have a higher likelihood of being observed. In this manner, each gene is assigned nine normalized gene scores for each CT, with the largest score informing the CT that is most likely the true CT for a gene’s expression dynamics.

### 3.7. KEGG Human Network (KEGG-HN)

In this study, our aim is to identify functionally related genes in metabolic and signaling pathways that share a similar dynamic differential gene expression profile when comparing RYGB vs. WM groups. We avoided traditional PEA to liberate the analysis from canonical pathway boundaries, and instead pursued module discovery on graph-based networks to account for interactions between genes that span multiple functions. Specifically, we developed a set of metrics to assess whether genes with the same high-scoring CTs cluster within sub-networks of a comprehensive large-scale network describing human metabolic and signaling pathways. Using the KEGG REST API [12] and Biopython package [24], the KGML files for all 337 KEGG human pathways were parsed for information regarding the interactions between genes and compounds. A network graph was constructed by abstracting these interactions with nodes denoting either genes or compounds, and edges describing their relationship, which can represent a metabolic reaction, a transcriptional regulation, or a protein–compound or protein–protein interaction. In this study, all edges were treated with equal weights despite there being different interaction types, metabolic reaction edges were annotated to account for reversible reactions, and the directionality of the edges were only used for visualization of networks, since our sub-network detection algorithm does not necessitate edges to be directed. The complete KEGG Human Network (KEGG-HN) is comprised of 5263 unique nodes and 19,615 edges, and is available in Supplemental Table S5, with the attributes of each node in Supplemental Table S6. These two files are compatible with Cytoscape [25] for visualization of the graph network.

### 3.8. Generating KEGG-HN Subnetworks

To identify groups of genes enriched with high normalized CT score for each CT, we applied the Monte Carlo sampling of subnetworks of KEGG-HN. Computationally, a sub-network is selected using the following procedure. First, a sub-network, S, is defined as an empty array of nodes. Next, a random node from KEGG-HN is chosen (N_1_) and added to subnetwork S. Then, a random edge is selected from all edges connected to N_1_, and the second node connected to the edge is added to sub-network S, where S is now [N_1_, N_2_]. Again, a random node is selected from S and a random edge is selected from all edges that are connected to that node. This node is added to S if it does not belong to S. This procedure is repeated until the subnetwork S has M protein nodes. If it takes more than 1000 iterations to add a new node, the procedure is terminated, and a new subnetwork is built starting from N_1_. The KEGG-HN was sampled for 100,000 sub-networks of size M, inclusively ranging from 10 to 20. Supplemental Table S2 contains the composition of each module.

### 3.9. Module Analysis

For each sub-network sampled, a module CT score is computed for each CT by taking the mean of the normalized CT scores across all genes in the sub-network. In this regard, modules with high modular CT score values are more likely to be enriched in genes that exhibit the CTs dynamic expression trajectory. Subnetworks are ranked by module CT scores.

To determine a significance threshold for the module CT sores, a null distribution of module CT scores is generated by shuffling the gene labels of our gene CT score dataset and recalculating module CT scores. This randomized dataset is designed to control for clustering of genes with high normalized CT scores for a given CT in sub-networks of KEGG-HN by random chance. The experimental and null cumulative distributions of module scores for each CT are compared using probability plots, elucidating that module scores observed for CTs 1–4 is significantly greater than those observed in the null model. Each module is then assigned a ρ value for each CT, calculated as the probability of observing a module CT score at least that high in the null distribution. For CTs 1–4, the group of modules with ρ equal to the minimum ρ observed are considered significant and are visualized in Cytoscape and further assessed for biological relevance.

## 4. Conclusions

We propose our computational platform as a universally applicable time-series omics analysis workflow that facilitates the discovery of network modules with common dynamic profiles. Focusing on RYGB surgery which triggers a complex, time-dependent, multi-organ metabolic and signaling rewiring [10], we report sub-networks of hepatic genes that may be partially responsible for the observed metabolic phenotype, which may have otherwise gone undetected with conventional pair-wise-omics analysis methods. While three time points are generally sufficient to capture an acute-phase, mid-point, and long-term impact of a perturbation, a natural extension of this work would enable any number of time points to be added, which would be critical in studying biological systems that exhibit more frequent fluctuations such as the circadian rhythm. Our treatment of trends as two vectors with both magnitude and direction, essentially acting as a four- parameter model, can be extended to more generalized functions that model time-course behavior without introducing additional parametrization. We have demonstrated here the need in omics-analysis for metabolism research to evolve and account for time-series data, which is imperative to the study of complex and dynamic metabolic disorders and the discovery of new druggable targets.

## Data Availability

The data presented in this study are available in article and Supplementary Material.

## Supplementary Materials

The following supporting information can be downloaded at: https://www.mdpi.com/article/10.3390/metabo12040318/s1. Figure S1: Trend frequencies when L2FC values are sampled from a normal distribution. Table S1: Gene data. Table S2: Sham data. Table S3: Kolmogorov Smirnov test. Table S4: Module data. Table S5: Human KEGG Network. Table S6: Human KEGG Attributes.
