# Supplementary material for: Rewiring of the Liver Transcriptome across Multiple Time-Scales Is Associated with the Weight Loss-Independent Resolution of NAFLD Following RYGB
Source: Metabolites. 2022 Apr 2;12(4):318. doi: 10.3390/metabo12040318 (PMC9025796; doi:10.3390/metabo12040318)
Supplement: Supplementary file 1 [file metabolites-12-00318-s001.zip › supplementary Figure S1.pdf]

SUPPLEMENTARY INFORMATION

Rewiring of the Liver Transcriptome Across Multiple Time-Scales is Associated with the Resolution of Fatty Liver Following RYGB

Peng Lei, Chijioke Chukwudi, Prabh R. Pannu, Shijie He, Nima Saeidi

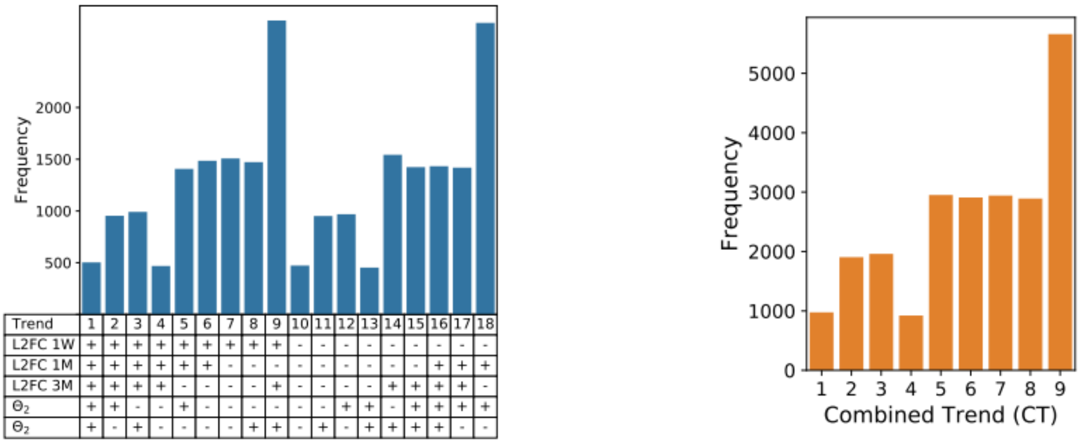

Trend	1	2	3	4	5	6	7	8	9	10	11	12	13	14	15	16	17	18
L2FC 1W	+	+	+	+	+	+	+	+	+	-	-	-	-	-	-	-	-	-
L2FC 1M	+	+	+	+	+	+	-	-	-	-	-	-	-	-	-	+	+	+
L2FC 3M	+	+	+	+	-	-	-	-	+	-	-	-	-	+	+	+	+	-
Θ_2	+	+	-	-	+	-	-	-	-	-	-	+	+	-	+	+	+	+
Θ_2	+	-	+	-	-	-	-	+	+	-	+	-	+	+	+	+	-	-

Supplementary Figure S1: Trend frequencies when L2FC values are sampled from a normal distribution.
